# Impact of occlusal reconstruction positions on airway dimensions in patients with edentulism and long centric occlusion

**DOI:** 10.1186/s12903-023-02931-1

**Published:** 2023-04-14

**Authors:** Huiying He, Sheng Zhang, Jun Xu

**Affiliations:** 1 Department of Stomatology, Beijing Tsinghua Changgung Hospital, School of Clinical Medicine, Tsinghua University, No.168 of Litang Road, Changping District, Beijing, 102218 China; 2 Department of Stomatology, Beijing Huairou Hospital, Beijing, 101400 China

**Keywords:** Airway, Centric relation position, Muscular position, Magnitude of long centric

## Abstract

**Objective:**

To study the airway changes of edentulous patients with a magnitude of long centric (MLC) ≥ 1.5 mm during occlusal reconstruction at the centric relation position (CRP) and muscular position (MP).

**Methods:**

The CRP and MP were determined by Gothic arch. The cephalometric analysis was taken at the two occlusal positions. The sagittal distance of each part of the upper airway was measured. The differences between two occlusal positions were compared. The difference values were calculated by subtracting the two. The correlation between the MLC and the difference value was analyzed.

**Results:**

The sagittal diameters of palatopharynx and glossopharynx airway at MP were statistically larger than those at CRP (*P* < 0.05). The MLC had a strong correlation with the ANB angle (*r* = 0.745, *P* < 0.001).

**Conclusion:**

Compared with the occlusal position of CRP, occlusion reconstruction at MP can provide better airway condition for edentulous patients with large MLC.

## Introduction

Determining the horizontal maxillomandibular relationship in complete denture restoration remains a topic of debate in the field of prosthodontics. Scholars have long believed that occlusion should be established in the only resettable position, namely, the centric relation position (CRP) [1, 2]. That is also called as centric position and is the physiological most retruded position of the mandible. However, with the progress of occlusal research, it has been proposed that the muscular position (MP), which is the terminal position of the muscular contraction path and relies on habitual opening and closing movement, better conforms to the physiological conditions [3–5]. MP in most patients is 0.5–1 mm ahead of CRP, and scholars call the distance between MP and CRP as “magnitude of long centric” (MLC) [4, 6, 7].

However, the MLC varies from person to person, and MP is affected by factor of head position, movement speed, and force, which is difficult to determine clinically [8, 9]. Previous studies have shown that if patients with a large difference between CRP and MP undergo occlusal reconstruction at CRP, their mandibles tend to move towards MP [10]. In patients with a large MLC, the occlusion constructed at the CRP or the position that 0.5–1 mm in front of the CRP may result in an unstable occlusion, denture sliding, or mucosal tenderness due to the excessive lateral force and the acceleration of the absorption of the residual alveolar ridge [10, 11]. A more unknown question worth investigating is whether the choice of occlusal position affects the airway, especially in the patients with large MLC. In recent year, scholors investigated the correlation between complete denture wearing and the upper airway dimentions [12, 13]. But there is no study to research the effect of different occlusal reconstruction positions when complete denture were made on airway in edentulous patients, so it’s meaningful and necessary. Therefore, we proposed a Hypothesis that different occlusal reconstruction positions would effect airway in patients. Herein, our study aimed to investigate the effect of different occlusal position on the airway of edentulous patients with an MLC of ≥ 1.5 mm during occlusal reconstruction of complete denture restoration, and further to provide a basis for determining the optimal method.

## Materials and methods

### Participants

Among the patients who were treated at the Department of Stomatology of Tsinghua Changgung Hospital in Beijing from 2016 to 2018, edentulous patients who would receive complete denture restoration and met the inclusion criteria were enrolled in this prospective study. All the participants provided a signed informed consent form. This study protocol was formulated in accordance with the requirements of the Declaration of Helsinki of the World Medical Association. It was approved by the Ethics Committee of Tsinghua Changgung Hospital in Beijing (NO 20150911–09).

The inclusion criteria were as follows: (1) edentulous patients with good alveolar ridge healing, (2) the difference between the CRP and the MP was ≥ 1.5 mm, and (3) patients with good compliance who were able to cooperate with clinical work.

The exclusion criteria were as follows: (1) patients with jaw dysplasia, (2) patients experiencing persistent pain in the oral and maxillofacial regions, (3) patients with temporomandibular joint disorder, and (4) patients with severe nasal obstructive disease.

### Research methods

#### Gothic arch tracing and measurement of the tapping point

All patients underwent Gothic Arch Tracing. The occlusion rims was used to determine the occlusal plane, midline and vertical dimention. The appropriate vertical dimension was determined by phonetic test. The Gothic arch (Geneva dental company, California) was placed routinely to ensure that the tracing plate was parallel to the occlusal plane and the bottom edge of the plate was perpendicular to the maxillary midline. The patient was instructed to sit upright and keep the head upright to allow for autonomous mandibular movement. It was ensured that both the forward extension and the lateral movement reached the maximum range and retreated to the rearmost position, and that each lateral movement started from the rearmost position. The vertex of the trace was regarded as the CRP and was subsequently marked, while the end point of each movement trace was also marked. The trace image was blackened except for each marked point. Ensuring that the vertical dimension remained unchanged, the device was placed into the patient's mouth again before the patient was instructed to sit upright while keeping their head straight and to perform habitual opening and closing movements 30 to 40 times. The center of the most concentrated area of the marks was selected as the terminal position of the muscular closing path, that is, the MP. A vernier caliper was used to measure the distance between the CRP and the MP along the direction of the anterior–posterior movement trajectory of the mandible, that is, the MLC. The measurement was conducted three times, with the average value taken as the result.

**Fig. 1:**
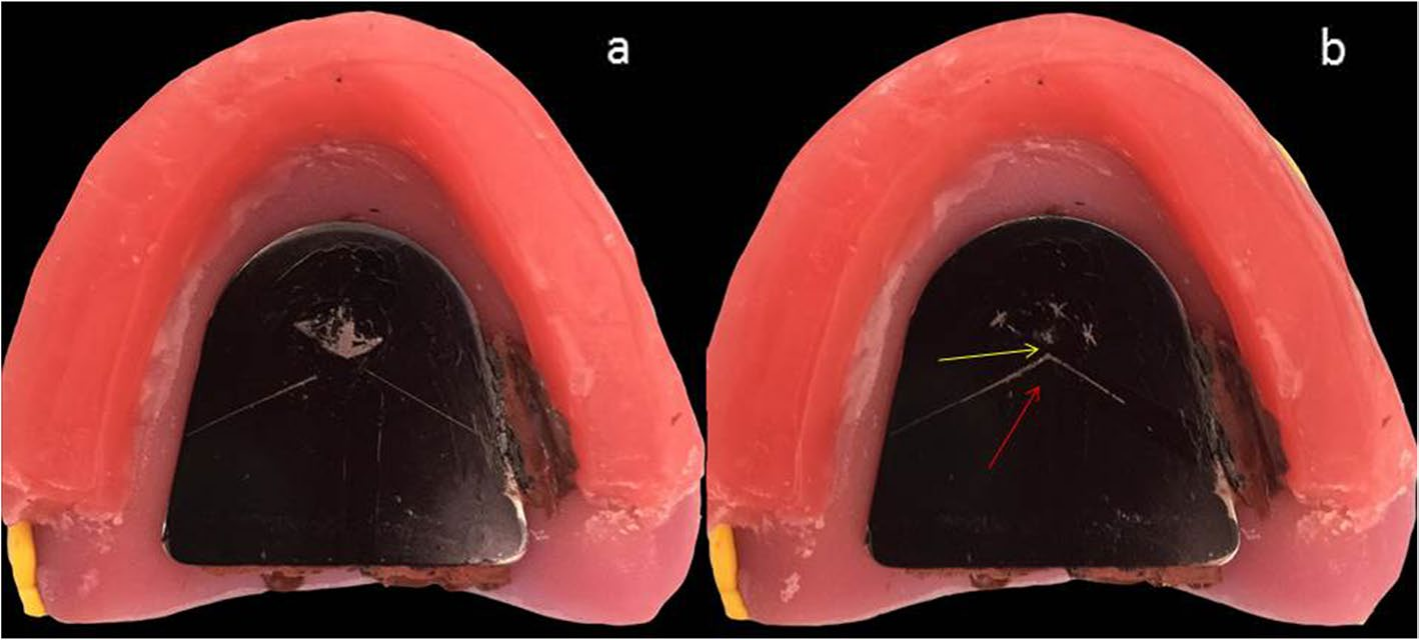
The gothic arch tracing and taping points of one patient. **a** The gothic arch tracing. **b** CRP and MP. The yellow arrow shows the tapping points, the center of these points is determined as the MP. The red arrow shows the CRP

The image of one patient’s gothic arch tracing and the CRP and MP points are shown in Fig. 1.

#### Lateral cephalometric analysis

**Fig. 2:**
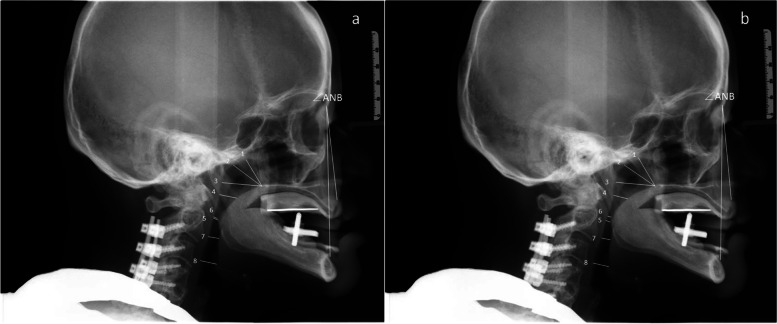
X-ray cephalometric lateral radiographs of the same patient as Fig. 1 at CRP and MP. **a** X-ray cephalometric lateral radiographs at MP. **b** X-ray cephalometric lateral radiographs at CRP. The numbers are measurement items of the upper airway as same as Fig. 3

The Gothic tracing needle was fixed in the CRP and the MP, respectively. The patients wore a denture base with a Gothic arch and X-ray cephalometric lateral radiographs were taken at both the CRP and the MP, with all the imaging performed by the same experienced technician using a Orthophos XG 3D camera (Sirona, Germany) (Fig. 2). The technical specifications for obtaining the standard cranial X-ray lateral images were as follows: (1) the patient stood upright, (2) the height of the positioning frame was adjusted, (3) earplugs were gently inserted into the ear canal on both sides, (4) the patient naturally relaxed their head with their eyes looking straight ahead, (5) the Frankfort horizontal plane was adjusted to be parallel to the ground, and (6) the patient breathed calmly without swallowing. The X-ray cephalometric lateral films were imported into a WinCeph 8.0 cephalometric analysis system, and important anatomical sites were traced and relevant data were measured.

#### Measurement items

**Fig. 3:**
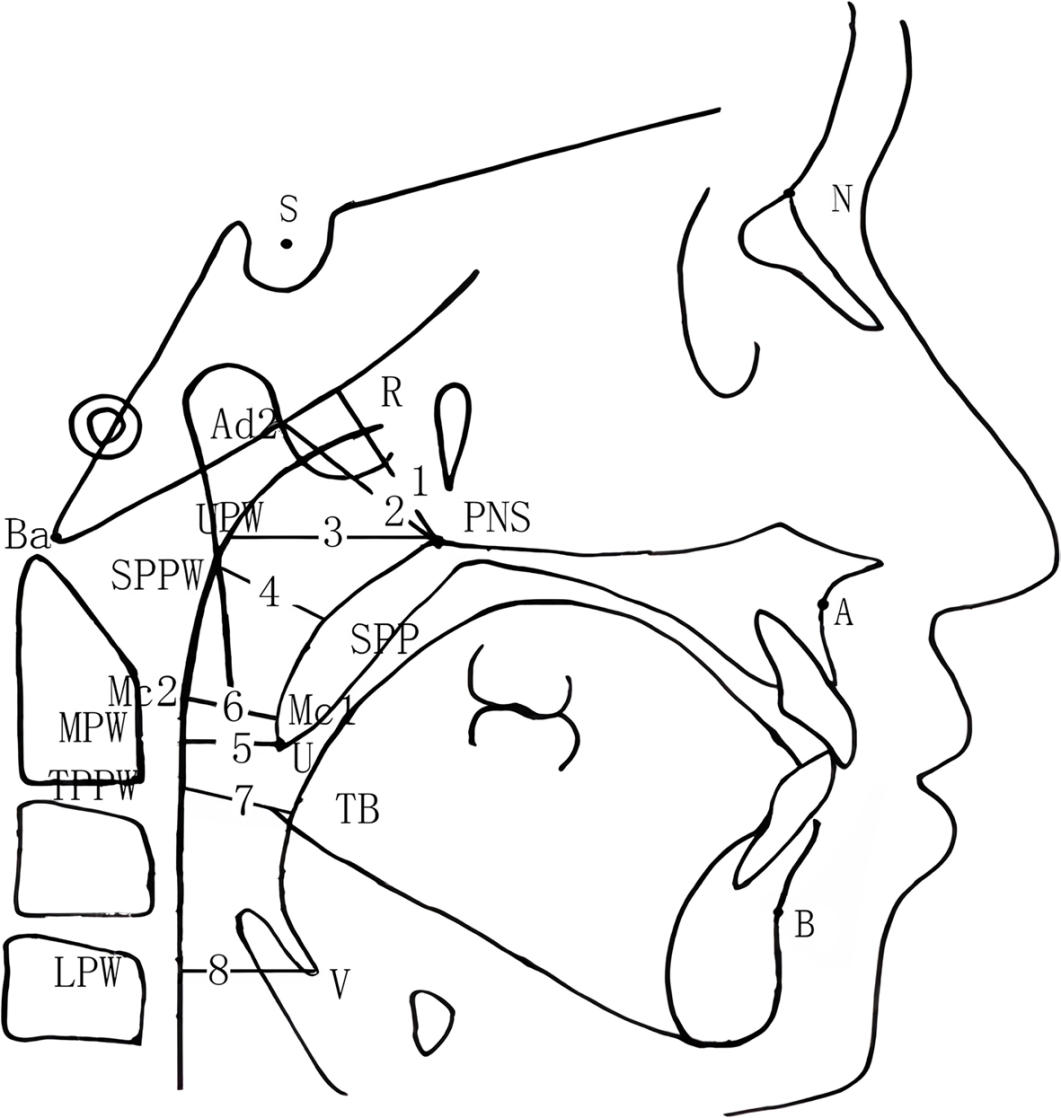
Measurement items of the upper airway. (1) The distance between the posterior nasal spine (PNS) and R point. (2) The distance between the PNS and the Ad2 point. 3) The distance between the PNS and the upper pharyngeal wall (UPW). (4) The distance between the soft palate posterior (SPP) surface and the soft palate posterior pharyngeal wall (PPW) (SPPW). (5) The distance between the uvula apex (U) and the middle pharyngeal wall (MPW). (6) The McNamara line (the distance between the Mc1 and Mc2 points). (7) The posterior airway space, which is the distance between the root of the tongue (TB) and the tongue PPW (TPPW). (8) The distance between the epiglottic vallecula (V) and the lower pharyngeal wall (LPW)

The measurement items were shown in Fig. 3.

##### Cephalometric landmarks

PNS: posterior nasal spine, the most posterior point on the sagittal plane of the hard palate on the mid-sagittal plane.

R: the intersection of the connecting line between the Ho point and the PNS with the posterior pharyngeal wall (PPW).

Ho: the intersection between the front edge of the lateral pterygoid plate and the lower edge of the posterior skull base.

S: the sella turcica point, a cephalometric landmark in the geometric center of the pituitary fossa of the sphenoid bone; a bony anatomic landmark.

Ba:the skull base point, the midpoint of the anterior edge of the foramen magnum.

Ad2: the intersection of the vertical line passing through the PNS of the connecting line between the sella turcica point(S) and the skull base point (Ba) with the PPW.

UPW: the upper pharyngeal wall point, the intersection of the PNS–Ba line with the PPW.

SPP: makes a vertical line to the PPW passing through the central point of the soft palate, the intersection of the the vertical line with the soft palate posterior surface.

SPPW: makes a vertical line to the PPW passing through the central point of the soft palate, the intersection of the the vertical line with the PPW.

U: uvula apex.

MPW: the middle pharyngeal wall point, which makes a vertical line to the PPW passing through point U, where the foot point is the MPW.

P: pogonion, the most anterior point on the mandible.

Id: infradentale, the uppermost point of the lower alveolar process.

B: supramentale, a landmark representing the deepest point of the symphyseal cavity between infradentale and pogonion.

Go: gonion, the outer point on either side of the lower jaw at which the jawbone angles upward.

TB: the intersection of the connecting line between the B and Go point with the root of the tongue.

TPPW: the intersection of the connecting line between the B and Go point with the PPW.

V: the epiglottic vallecular, a depression just behind the root of the tongue between the medial and lateral glosso-epiglottic folds in the throat.

LPW: the lower pharyngeal wall point, a vertical line passing through point V to the PPW, where the foot point is the LPW.

SPr: superior prosthion, the anterior-lower point of the upper alveolar process.

A: subspinale, the most concave point of bone between anterior nasal spine and SPr.

N: nasion, a bony cephalometric landmark at which the nasofrontal suture is bisected by the midsagittal plane.

##### Cephalometric measurements

Nasopharynx

- (1) PNS-R: The distance between PNS and R.
- (2) PNS-Ad2: The distance between PNS and Ad2.
- (3) PNS-UPW: The distance between PNS and UPW.

Palatopharynx

- (4) SPP-SPPW: The distance between SPP and SPPW.
- (5) U-MPW: The distance between U and MPW.
- (6) Mc1 —Mc2: the minimum distance between the soft palate posterior surface and the PPW.

Glossopharynx

- (7) TB-TPPW: The posterior airway space, the distance between TB and TPPW.
- (8) V-LPW: The distance between the V and LPW.

ANB angle: in cephalometric analysis, the angle formed between the nasion point A line and the nasion point B line.

Each measurement was repeated twice by one investigator, the second measurement was made about one hour later, and the average value was taken as the result in each case.

### Statistical methods

The data were statistically analyzed using SPSS™ Statistics v22.0 software (IBM,USA). The values taken from the patients with occlusal positions at the CRP and the MP were compared using the paired sample *t*-test, with the difference values obtained by subtracting the measured values of the upper airway based on the occlusal position at the MP from those based on the CRP. The correlation between the MLC and the difference value was then analyzed by Pearson correlation analysis.

## Results

Twenty-one edentulous patients who would receive complete denture restoration and met the inclusion criteria were enrolled in this prospective study. The group comprised 11 males and 10 females, with an average age of 74.1 years (range from 60 to 86 years).

The sagittal distance of the palatopharynx and glossopharynx airway in patients at MP were statistically larger than those at the CRP (*P* < 0.05). There was no significant difference in the sagittal distance of the nasopharynx airway between the two occlusal positions (*P* > 0.05) (Table 1).

**Table 1:** Comparison of measurement items between CRP and MP (mm,$\overline{{\text{x}}}$ ± SD)

Measurement item	CRP	MP	P
PNS-R	28.21 ± 2.67	28.57 ± 2.74	0.65
PNS-Ad2	29.09 ± 3.34	29.29 ± 2.77	0.535
PNS-UPW	28.97 ± 2.25	29.08 ± 2.28	0.7
Mc1-Mc2	3.11 ± 2.03	4.50 ± 2.85	0.000*
SPP-SPPW	10.01 ± 2.70	10.93 ± 2.73	0.19*
U-MPW	5.20 ± 2.84	6.94 ± 3.55	0.000*
TB-TPPW	5.88 ± 2.41	7.58 ± 3.75	0.000*
V-LPW	12.34 ± 5.12	13.89 ± 6.41	0.002*
ANB	30.24 ± 40.65	17.81 ± 43.52	0.000*

Centric relation position (CRP), muscular position (MP)

*: *P* < 0.05

The MLC had a strong correlation with the ANB angle (*r* = 0.745, *P* < 0.001). There were no correlation between the MLC and the changes in the other measured parameters (*P* > 0.05).

## Discussion

Many previous studies have shown that there was a difference between MP and CRP in about 90% of people, most of which were 0.5 to 1.0 mm in ahead of CRP [3, 6, 7, 10]. However, the measurement results for the MLC in edentulous patients obtained by various scholars have been largely inconsistent. The measurement of Boos [6] and Lammie et al. [3] was 0.5 mm, and the measurement of Posselt was 1.25 ± 1.00 mm [7]. Li et al. [10] measured 1.02 ± 0.36 mm, and Liu et al. [11] measured 0.39–0.99 mm. This inconsistency could be a bias due to differences in sample size, but it's clear that MLC varies from person to person.

In the previous studies conducted by this manuscript’s research team, the detection rate of patients with a large MLC (≥ 1.5 mm) was around 10% to 20% [10, 11]. For this part of patients, the occlusal position should theoretically be established at the MP that most conforms to the normal physiological conditions. However, given that the MP is not the only stable position, it is easily affected by various factors, such as the position, muscle state, and mental state of patients, which is difficult to determine the position clinically [8, 9]. Therefore, a number of researchers have suggested establishing the occlusal position at the classic CRP.

To date, the main concerns regarding the occlusal position have included the influence of lateral force on denture retention and the stability and resorption of the remaining alveolar bone. For certain, it is important to consider the impact of different occlusal positions on the adjacent tissues of the oral cavity and whether this would change the airway and thereby affect the breathing. Therefore, this study was the first to investigate the impact of occlusal position from the perspective of airway.

The study results revealed that, for edentulous patients with an MLC of ≥ 1.5 mm, the mandible was positioned slightly forward at the MP, and the sagittal distance of the upper airway palatopharynx and glossopharynx were larger. However, when the mandible was at the CRP, it was positioned in a more backward direction, and the sagittal distance of the upper airway palatopharynx and glossopharynx were smaller. The difference in the change of the distance of the nasopharynx was not statistically significant since the nasopharynx was mostly surrounded by bony structures and was less affected by any positional changes of the mandible.

These results were similar to the changes in the upper airway after changing the mandibular position in patients with dentition issues [14–17]. Previous studies have revealed that mandibular advancement could enlarge the upper airway [14–17], which was manifested in an increase of the sagittal and transverse distance of the upper airway and its resulting change in shape. Meanwhile, moving the mandible backward could bring the base of the tongue closer to the PPW and narrow the upper airway [18–22].

Upper airway stenosis had also been found to be closely related to obstructive sleep apnea hypopnea syndrome (OSAHS), a medical disorder characterized by recurrent respiratory arrest and upper airway obstruction during sleep [23]. OSAHS patients experience apnea and decreased blood oxygen saturation. In the long term, OSAHS may lead to various complications, such as heart, brain, and kidney diseases, and can even result in sudden death.

A previous study [24] confirmed that the upper airway space of patients with OSAHS is significantly smaller than that of healthy individuals and is concentrated at all levels of the oropharynx. Furthermore, the area of the tongue and the soft palate tends to be larger and the remaining area of the oropharynx smaller, meaning the pathogenesis of OSAHS is closely related to the shape abnormalities of the upper airway and the surrounding structures, including the stenosis of the upper airway space in the oropharynx.

Previous studies showed that move the mandibula could change airway dimensions by setting the mandibular advancement to the edge or even the maximum advancement position in patients with OSAHS [16], performing orthodontic or surgical treatment of mandibular advancement in patients with mandibular hypoplasia [17, 25], or performing mandibular setback surgery in patients with mandibular prognathism [19–22]. The changes in the mandible movement in the above studies were large at over ten millimeters or even tens of millimeters. In addition to the anterior-to-posterior changes, there often emerged vertical changes in the process of advancement and setback, and orthodontic or surgical procedures to move the mandible forward or backward did not present autonomous changes under normal physiological conditions.

However, in the present study, the airway changes in the edentulous patients were investigated in terms of two physiological occlusal positions in the same vertical dimension. Here, the change in the distance between the two occlusal positions was relatively small (only several millimeters), and the movement of the mandibular setback from the MP to the CRP, which was the physiological rearmost position, was autonomous. As such, the focus of this study was different from those in the previous studies.

The results of the present study revealed that the sagittal distance of the palatopharynx and glossopharynx at the CRP were smaller than those at the MP, while the palatopharynx was the narrowest part of the upper airway. If the occlusal position was established at the CRP, the sagittal distance at the narrowest point would be further reduced, while it was unclear whether this would cause the reduction of ventilation to induce OSAHS or other complications. However, in comparison, establishing the occlusal position at the MP could increase the sagittal distance of the palatopharynx and glossopharynx and could improve the stenosis of the upper airway, thereby reducing the occurrence of the aforementioned issues. Previous studies involving patients with OSAHS have also confirmed this finding. Kato et al. [26] observed that the nocturnal blood oxygen saturation in their patients increased proportionally during mandibular advancement by 2, 4, and 6 mm, and that the improvement in breathing was linearly related to the mandibular advancement. Therefore, for patients with a large MLC, to improve the breathing, it is recommended that the occlusal position is established at the MP.

In terms of other factors, if occlusion of the complete denture is established at the CRP, when the patient habitually bites, it will deviate from the CRP. And the functional cusp will bite on the inclined surface of the central fossa instead of on the bottom of the central fossa during mastication. Those will result in a lateral force that could destroy the stability of the denture and could even lead to alveolar bone resorption [10, 11]. This further suggested that the occlusal position should be established at the MP. Meanwhile, the issue of determining the MP clinically could be resolved by applying the modified occlusal pattern to increase the tolerance of mesial and distal direction to achieve freedom of centric [10].

According to the results of previous studies [14–16, 26–28], following mandibular advancement, the sagittal distance of the airway increased, and the transverse distance of each airway segment increased to varying degrees. The increase in transverse distance exceed the sagittal distance, and the shape of the upper airway gradually changed from an ellipse with a longer transverse distance and a smaller sagittal distance to a more transversely flat ellipse, thereby improving the stenosis of the upper airway and increasing ventilation. Since the sagittal distance results obtained in the current study were similar to those obtained in previous studies.

In this study, the possible correlation between the MLC and the changes in the sagittal distance of various parts of the airway was explored. The results revealed that there was no correlation between the MLC and each difference value of the sagittal distance of the airway. Tsuiki et al. [29] found that during the process of mandibular advancement by 33%, 67%, and 100%, the upper airway measurement index on the X-ray cephalometric lateral radiographs exhibited a gradual but disproportionate increase. Elsewhere, Gao et al. [30] adopted magnetic resonance imaging technology and found that during the process of mandibular advancement by 0%, 50%, 75%, and 100%, the size of the upper airway consistently increased, while from the 75% point on, the increase tended to be more gradual. This was likely because the airway is a three-dimensional structure, and in addition to the sagittal distance, changes in the transverse distance and geometry also led to the same result.

One of the limitations was that the small sample size and single center prospective study may weaken the generalisability of the results. Besides, the X-ray cephalometric lateral imaging method can only form two-dimensional plain images, but cannot obtain three-dimensional results, which affects the comprehensiveness of the results. It has been confirmed that three dimensional methods of the airway were more reliable and precise phrasing of a statement of postoperative gradients than conventional radiography [17]. Due to the metal artifacts produced by cone beam computed tomography (CBCT) method when gothic arch was used, the classical method of airway observation, X-ray cephalometric lateral imaging, was chosen in this study. If non-metallic Gothic arch could be produced in the future, it will be very meaningful to use CBCT to measure the three-dimensional changes of the airway. The other limitation was that measurements were not performed by a second rater, Inter-Rater-Reliability was not investigated. Finally, it is known, that the position of the patient inside the gantry has an effect on the projection of the posterior airway [31, 32]. As most of the manufacturer instructions for taking lateral radiographs do not describe the required stance of the patient inside the gantry, there is a certain leeway space for different alignment within the gantry [31, 32]. Future studies have to be conducted to investigate if there are inaccuracies caused by this effect.

## Conclusion

Compared with the occlusal position of CRP, occlusion reconstruction at the MP point can provide better airway condition for edentulous patients with large magnitude of long centric.

## Data Availability

The datasets used and/or analysed during the current study available from the corresponding author on reasonable request.

## Abbreviations

CRP: Centric relation position
MP: Muscular position
TMJ: Temporomandibular join
OSAHS: Obstructive sleep apnea hypopnea syndrome
CBCT: Cone beam computed tomography
MLC: The magnitude of long centric
